# Comparative performance of the Platelia *Aspergillus* Antigen and *Aspergillus* Galactomannan antigen Virclia Monotest immunoassays in serum and lower respiratory tract specimens: a “real-life” experience

**DOI:** 10.1128/spectrum.03910-23

**Published:** 2024-06-25

**Authors:** Eliseo Albert, María Jesús Alcaraz, Estela Giménez, María Ángeles Clari, Ignacio Torres, Javier Colomina, Beatriz Olea, Mar Tormo, José Luis Piñana, Rosa Oltra, Jaime Signes-Costa, Nieves Carbonell, Carlos Solano, David Navarro

**Affiliations:** 1 Microbiology Service, Clinic University Hospital, INCLIVA Health Research Institute, Valencia, Spain; 2 Hematology Service, Clinic University Hospital, INCLIVA Health Research Institute, Valencia, Spain; 3 Infectious Diseases Unit, Clinic University Hospital, INCLIVA Health Research Institute, Valencia, Spain; 4 Pulmonary Department, Clinic University Hospital, INCLIVA Health Research Institute, Valencia, Spain; 5 Medical Intensive Care Unit, Clinic University Hospital, INCLIVA Health Research Institute, Valencia, Spain; 6 Department of Medicine, School of Medicine, University of Valencia, Valencia, Spain; 7 Department of Microbiology School of Medicine, University of Valencia, Valencia, Spain; 8 CIBER de Enfermedades Infecciosas, Instituto de Salud Carlos III, Madrid, Spain; Foundation for Innovative New Diagnostics, Geneve, Switzerland

**Keywords:** Aspergillus, galactomannan, invasive pulmonary aspergillosis, Platelia immunoassay, Virclia immunoassay

## Abstract

The Platelia Aspergillus Antigen immunoassay is the “gold standard” for Aspergillus galactomannan (GLM) measurement in sera and bronchoalveolar lavage (BAL) for the diagnosis of invasive pulmonary aspergillosis (IPA). We evaluated the performance of the Aspergillus GLM antigen Virclia Monotest compared to the Platelia assay. A total of 535 specimens [320 sera, 86 bronchial aspirates (BAs), 70 BAL, and 59 tracheal aspirates (TAs)] from 177 adult patients (72 hematological, 32 Intensive Care Unit, and 73 hospitalized in other wards) were processed for GLM testing upon clinical request. One patient had proven IPA, and 11 had probable disease. After excluding indeterminate Virclia results (*n* = 38), 396 specimens yielded concordant results (56 positive and 340 negative) and 101 discordant results (Virclia positive/Platelia negative, *n* = 95). The overall agreement between immunoassays was higher for sera (*κ* 0.56) than for BAL (*κ* ≤ 0.24) or BAS and TA (*κ* ≤ 0.22). When considering all specimen types in combination, the overall sensitivity and specificity of the Virclia assay for the diagnosis of proven/probable IPA were 100% and 65%, respectively, and for the Platelia immunoassay, sensitivity and specificity were 91.7% and 89.4%, respectively. The correlation between index values by both immunoassays was strong for serum/BAL (*ρ* = 0.73; *P* < 0.001) and moderate for BAS/TA (Rho = 0.52; *P* = 0.001). The conversion of Virclia index values into the Platelia index could be derived by the formula *y* = (11.97 * *X*)/3.62 + *X*). Data from GLM-positive serum/BAL clinical specimens fitted the regression model optimally (*R*^2^ = 0.94), whereas that of BAS and TA data did not (*R*^2^ = 0.11). Further studies are needed to determine whether the Virclia assay may be an alternative to the Platelia assay for GLM measurement in sera and lower respiratory tract specimens.

**IMPORTANCE:**

Galactomannan detection in serum or bronchoalveolar fluid specimens is pivotal for the diagnosis of invasive pulmonary aspergillosis (IPA). The Platelia Aspergillus Antigen immunoassay has become the “gold standard” for Aspergillus GLM measurement. Here, we provide data suggesting that the Virclia Monotest assay, which displays several operational advantages compared with the Platelia assay, may become an alternative to the Platelia assay, although further studies are needed to validate this assumption. We also provide a formula allowing the conversion of Virclia index values into Platelia values. The study may contribute toward positioning the Virclia assay within the diagnostic algorithm of IPA.

## INTRODUCTION

Diagnosis of proven invasive pulmonary aspergillosis (IPA) requires histological or cytopathological evidence of fungal tissue invasion or recovery of *Aspergillus* spp. by culture or detection by polymerase chain reaction (PCR) from a normally sterile site by biopsy or needle aspiration from a lesion consistent with an infectious process (1, 2). A wide variety of non-culture-based methods including detection of *Aspergillus* galactomannan (GLM) or β-d-glucan and Aspergillus-specific DNA by PCR are widely used across centers (3) for supporting a probable IPA diagnosis (4). *Aspergillus* spp. GLM is a cell wall polysaccharide that is released in tissues during hyphal development and behaves as a surrogate biomarker of fungal invasivity (5). The clinical usefulness of GLM detection in serum or bronchoalveolar fluid (BAL) specimens for the diagnosis of IPA has been shown both in hematological and intensive care unit (ICU) patients (1, 6–9). Since its FDA clearance in 2003, the Platelia Aspergillus Antigen immunoassay (Bio-Rad, CA, USA) (10–12), marketed as a sandwich enzyme-linked immunosorbent assay microtiter plate, has become the “gold standard” for Aspergillus GLM measurement. To avoid incurring high laboratory-associated costs, batch testing is routine practice in many laboratories using the Platelia assay, which may translate into delayed result reporting. Several lateral flow assays (LFA) capable of detecting *Aspergillus* antigen (including GLM) in serum and BAL within the hour are commercially available. Overall, the clinical diagnostic efficacy of LFAs performed in BAL specimens for proven/probable IPA has been reported to be good in virus-associated IPA (13), although may underperform in other risk population groups compared to ELISA-based assays such as the Platelia test (14–17). A new chemiluminescence-based immunoassay, the Aspergillus GLM antigen Virclia Monotest (Vircell S.L., Granada, Spain) has been recently marketed for GLM quantification in sera and BAL specimens. The operational advantages of this immunoassay compared with the Platelia assay include its monotest format, and automated processing in a random-access platform, which makes it possible to provide “same day” results (in around 1 h) at a relatively low cost. A few studies, mainly including hematological patients, have compared the analytical and clinical performance of both immunoassays (18–20). These studies uniformly showed that, from a qualitative standpoint, the Virclia assay performs at least comparably to the Platelia assay when using sera and BAL specimens. Moreover, the Virclia immunoassay provided quantitative values in sera and BAL that correlated reasonably well with those returned by the Platelia assay (18–20). Hence, the Virclia immunoassay shows promise as to its potential use as an alternative to the Platelia assay for the diagnosis of IPA. Here, we further compared the performance of both GLM immunoassays using consecutive non-cryopreserved serum and lower respiratory tract (LRT) specimens, including BAL, bronchoscopic aspirates (BAs), and tracheal aspirates (TAs), which were prospectively collected from hematological and non-hematological patients and tested in parallel upon clinical request. Moreover, we evaluated whether quantitative values returned by the Virclia immunoassay could be reliably converted into Platelia index values over the linear dynamic range of the latter assay; this is of clinical relevance provided that the Platelia GLM index values, both in serum and BAL, above clinically validated cut-offs, have been established as mycological criteria for the diagnosis of IPA in current consensus guidelines (1, 6–9).

## MATERIALS AND METHODS

### Participants and specimens

In this observational, single-center study, a total of 535 specimens (320 sera, 70 BAL, 86 BAS, and 59 TA) were prospectively collected from 177 consecutive adult patients (111 males/66 females; median age, 62 years; IQR, 51–70) between 12 January and 31 May 2023, and processed for GLM testing upon clinical request (Table 1). A total of 296 specimens were collected from 72 hematological patients, 139 from 32 ICU patients, and the remaining 100 from 73 patients hospitalized in internal medicine, pneumology, nephrology, or oncology wards. A total of 65 patients had a single specimen collected, whereas the remaining 112 patients had two or more (Table S1).

**TABLE 1:** Demographic and clinical features of patients included in the study

Parameter	No. (%)
Gender	
Male	111 (62.7)
Female	66 (37.3)
Clinical Unit	
Hematology	72 (40.7)
Pneumology	47 (26.5)
Critical Care Unit	32 (18.1)
Oncology	9 (5.1)
Internal Medicine	5 (2.8)
Neurology	3 (1.7)
Nephrology	1 (0.5)
Other	8 (4.5)
Underlying conditions in hematological patients	
Allogeneic hematopoietic stem cell transplantation	37 (20.9)
Lymphoma	14 (7.9)
Acute/chronic leukemia	13 (7.3)
Multiple myeloma	3 (1.6)
Other	5 (2.8)
Categorization of Invasive Aspergillosis^*a*^	
Proven	1 (0.5)
Probable	11 (6.2)
Possible	33 (18.6)
Discarded	132 (74.7)

^*a*^ Patients were categorized according to the 2020 European Organization for Research and Treatment of Cancer (EORTC)/Mycoses Study Group Education and Research Consortium (MSGERC) as having proven, probable, possible, or no IPA (1). COVID-19-associated pulmonary Aspergillosis in ICU patients (CAPA) and Influenza-associated pulmonary aspergillosis in ICU patients (IAPA) were defined according to consensus criteria for research and clinical guidance (13, 14). Neither the results of Platelia nor Virclia assays were considered as a mycological criterium for Invasive Aspergillosis.

### GLM immunoassays

Before testing, to dissociate immune complexes and precipitate proteins, serum and BAL samples were pretreated with an EDTA solution (provided in the Platelia kit) for 6 min in a heat block at 120°C and then centrifuged at 10,000 *g* for 10 min, following the manufacturer’s recommendations. BAS and TA samples with high viscosity were pretreated with the lowest possible amount of dithiothreitol (Sputasol, Oxoid Ltd., Basingstoke, United Kingdom) to obtain a homogeneous liquid consistency and were then processed similarly to BAL and sera. The supernatants were used for GLM testing. All samples were tested in parallel with the Platelia and Virclia assays in singlets within 48 h of receipt. The specimens were kept at 4°C until testing. The Platelia immunoassay was carried out manually. The threshold for positivity was set at an index value of ≥0.5 for sera, following the manufacturer’s recommendation, and ≥0.5 or ≥1.0 for BAL specimens, as either suggested by the manufacturer or due to its wide use in clinical practice, respectively (2–6). For analysis purposes, we also used ≥0.5 and ≥1.0 cut-offs for BAS and TA. The GLM Ag Virclia MONOTEST, which has been validated for sera and BAL specimens, was performed on a VirClia autoanalyzer. A calibrator control included in the kit was used to calculate the sample index value [Relative Light Units (RLUs) sample/RLU calibrator]. Index values >0.2 were considered positive for all specimens tested, between 0.16 and 0.20 were categorized as indeterminate, and those <0.16 were considered negative, in all instances following the manufacturer’s recommendations.

The Aspergillus GLM VIRCLIA sample set VSP001 (Vircell SA., Granada, Spain) consisting of 24 human sera, 10 negative and 14 with known GLM concentrations ranging from 0.075 to 1 µg/mL (Table S2) was employed to assess the correlation between index values returned by both GLM assays in different matrices, as indicated, and to determine whether a conversion factor between index values for both immunoassays could be derived. For analysis purposes, index values were rounded to two decimal places.

### Other mycological procedures

LRT samples were assessed by direct microscopy and cultured in Sabouraud agar following local practices. Aspergillus PCR testing in LRT specimens was performed when indicated using the AspID multiplex PCR assay, which is designed to detect genomic DNA of clinically relevant *Aspergillus* spp. (OLM Diagnostics, UK), and interpreted following the manufacturer’s recommendations.

### Definitions

Non-ICU patients were categorized according to the 2020 European Organization for Research and Treatment of Cancer (EORTC)/Mycoses Study Group Education and Research Consortium (MSGERC) as having proven, probable, possible, or no IPA (1, 9). ICU patients were categorized according to recent criteria published by Bassetti *et al*. (2). COVID-19-associated pulmonary Aspergillosis in ICU patients (CAPA) and Influenza-associated pulmonary aspergillosis in ICU patients (IAPA) were defined according to consensus criteria for research and clinical guidance (21, 22). To evaluate the clinical performance of the two GLM immunoassays, the results of the Platelia assay were not considered a mycological criterium for IA, CAPA, or IAPA, following the approach of Buil *et al.* (20). This aimed to avoid the introduction of a bias skewed toward an outperformance of the Platelia assay. Likewise, the results of the Virclia assay were not taken into consideration for patient categorization. Moreover, indeterminate results returned by the Virclia assay were discarded for analysis purposes. To evaluate the clinical performance of the GLM immunoassays, patients were classified as concordant when both the Platelia and Virclia assays returned one or more positive results, irrespective of the sample type and whether or not these were obtained for the same specimen, or when both GLM immunoassays yielded negative results over the study period. In turn, patients were categorized as discordant when one of the two immunoassays returned one or more positive results regardless of the sample type and the other systematically yielded negative results; for these analyses, the Platelia index cut-off value chosen for positivity in LRT was ≥0.5.

### Statistical analysis

Positive and negative percentage agreement (PPA and NPA) values are reported throughout the study. The level of agreement between the results returned by the assays was assessed by Cohen’s kappa statistics. *κ* values ≥ 0.7 were deemed as representing a high-level agreement. The clinical sensitivity and specificity of the GLM immunoassays were calculated only considering proven and probable IPA cases, thus excluding cases of possible IPA, and patients with no IPA as controls. Statistical comparisons were carried out by the McNemar test. Receiver operating characteristic (ROC) curves were built to assess the diagnostic efficiency of the Virclia and Platelia assays for probable/proven vs. no IPA. The McNeil test was used for Area under the ROC curves (AUC) value comparisons. The Spearman rank test was used to evaluate the correlation between quantitative values returned by both immunoassays; correlation coefficient interpretation was regarded as negligible (0.00–0.10), weak (0.10–0.39), moderate (0.40–0.69), strong (0.70–0.89), and very strong (0.90–1.00). The coefficient of determination (*R*^2^) was calculated to assess the proportion of variation in the dependent variable (Virclia index value) that is predictable from the independent variable (Platelia index value). Two-sided exact *P* values were reported. A *P*-value < 0.05 was considered statistically significant. The analyses were performed using SPSS version 20.0 (SPSS, Chicago, IL, USA). Graphical design was performed using GraphPad Software Inc v6.0 (California, USA).

## RESULTS

### Patient population

After excluding GLM results as a mycological criterium for IPA, of the 177 patients included in the study, only 1 had proven IPA (a hematological patient), 11 had probable disease (2 CAPA and 3 IAPA in ICU patients), and 33 had possible disease (all hematological patients). IPA was deemed absent in the remaining 132 patients. *Aspergillus fumigatus* was recovered from BAL cultures of seven patients with probable IPA (five hematological and two ICU patients). The remaining four patients with probable disease tested positive via PCR (*A. fumigatus*) but negative by culture.

### Qualitative agreement between results returned by the GLM immunoassays

**FIG 1:**
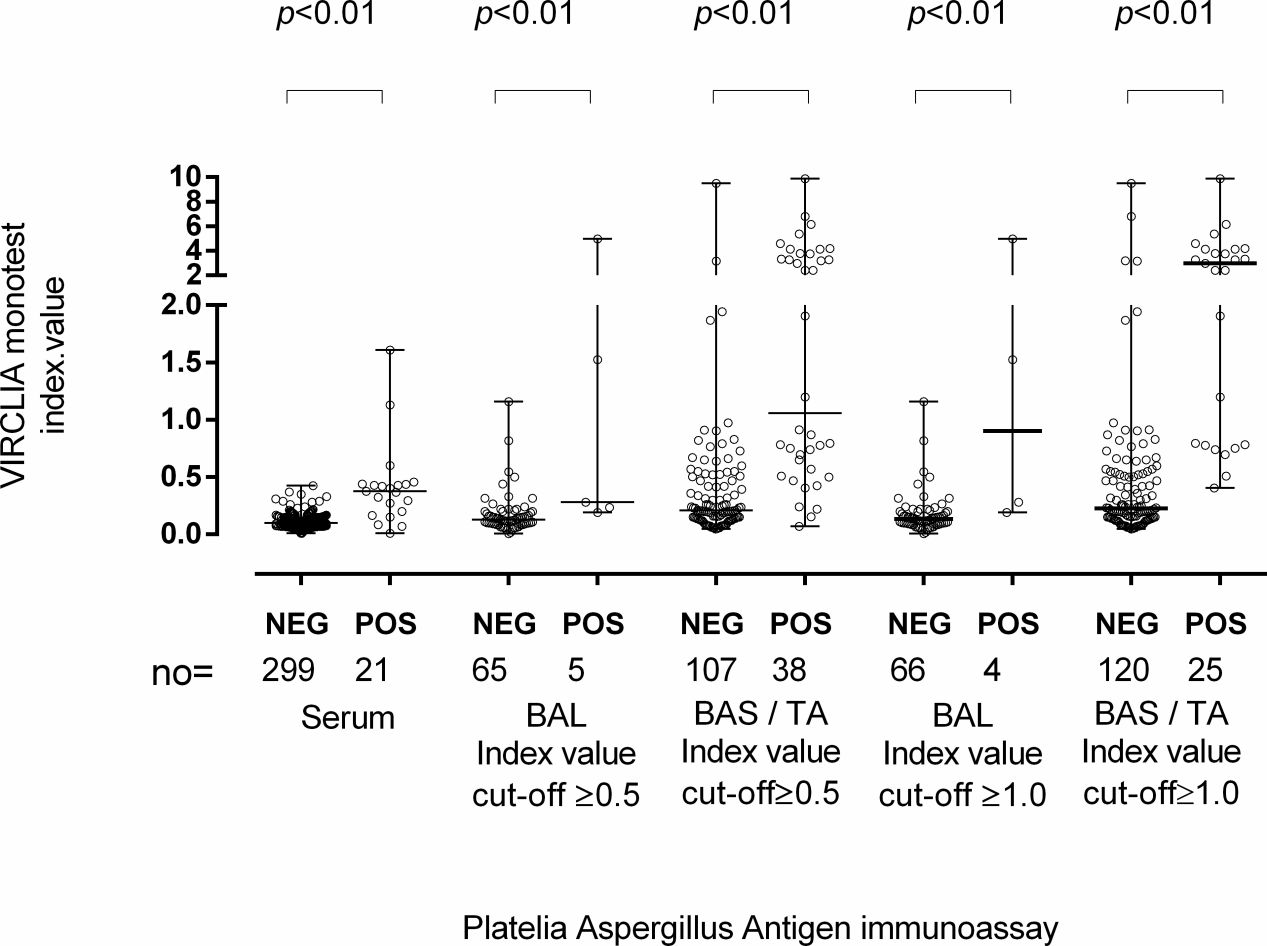
Index values returned by the Virclia immunoassay for samples testing either positive or negative with the Platelia immunoassay. BAL refers to bronchoalveolar lavage specimens. *P* values for comparisons are shown.

As shown in Table 2, overall, of the 535 specimens, the Virclia assay returned 151 positive, 346 negative, and 38 indeterminate results (21 sera, 7 BAL, 8 BAS, and 2 TA). In turn, when the positivity cut-off was set at ≥0.5, following the manufacturer’s recommendation, the Platelia assay yielded 64 positive and 471 negative results. After excluding the specimens returning indeterminate results with the Virclia assay, and considering an index value ≥0.5 for Platelia positivity, a total of 396 specimens yielded concordant results (56 positive and 340 negative) and 101 returned discordant results, yet the majority of which tested Virclia positive/Platelia negative (*n* = 95) and a few Platelia positive/Virclia negative (*n* = 6). Accordingly, the overall PPA across assays was low (36%), the NPA high (77%), and the Kappa index low (0.42; 95% CI, 0.32–0.52). We then compared the qualitative performance of both GLM immunoassays according to sample type. For all specimen types, the Virclia assay returned a substantially higher number of positive results compared with the Platelia assay (Table 2). In fact, when considering the ≥0.5 index value cut-off for the Platelia assay, only 37% of specimens testing positive with the Virclia assay, did so with the Platelia assay; the figures were 47% for sera, 43% TA, 32% BAS, and 20% BAL. For all specimen types, median index values returned by the Virclia assay were significantly lower in those yielding negative Platelia results compared with those from samples returning positive results (Fig. 1). As shown in Table 3, the best PPA value was found for sera (42%), followed by TA, BAS, and BAL. In turn, the NPA value was highest for sera (92%), followed by BAL, BAS, and TA. Regardless of the sample type, PPA values were very low compared with NPA values. Importantly, the overall agreement between immunoassays as qualified by Kappa statistics was substantially higher for sera (0.56; 95% CI, 0.38–0.73) than for BAL (≤0.24) or BAS and TA (≤0.22).

**TABLE 2:** Qualitative agreement between results returned by the Platelia Aspergillus Antigen assay and Aspergillus GLM antigen Virclia Monotest according to the sample type

Aspergillus GLM antigen Virclia Monotest result according to the sample type (no. of specimens)^*a*^	Platelia Aspergillus Antigen assay result^*b*^	
	Positive (for lower respiratory tract specimens ≥ 1/≥0.5)	Negative (for lower respiratory tract specimens <1/<0.5)
All specimens (535)		
Positive (151)	44/56	107/95
Negative (346)	4/6	342/340
Indeterminate (38)	1/2	37/36
Sera (320)		
Positive (34)	16	18
Negative (265)	4	261
Indeterminate (21)	1	20
Bronchoalveolar lavage (70)		
Positive (21)	3/4	18/17
Negative (42)	0/0	42/42
Indeterminate (7)	1/1	6/6
Off-label respiratory specimens (145)		
Positive (96)	25/36	71/60
Negative (39)	0/2	39/37
Indeterminate (10)	0/0	10/10
BAs (86)		
Positive (52)	11/17	41/35
Negative (26)	0/1	26/25
Indeterminate (8)	0/0	8/8
TAs (59)		
Positive (44)	14/19	30/25
Negative (13)	0/1	13/12
Indeterminate (2)	0/0	2/2

^*a*^ Aspergillus GLM antigen Virclia Monotest results: positive values ≥0.2; negative values <0.2; Indeterminate values between 0.16 and 0.19.

^*b*^ Platelia Aspergillus Antigen Assay result: Positive ≥0.5 in serum samples and either ≥0.5 or ≥1 in lower respiratory tract specimens.

**TABLE 3:** Agreement between the qualitative results returned by the Platelia Aspergillus Antigen and Aspergillus GLM antigen Virclia Monotest according to sample type

Sample type	Positive percentage agreement %	Negative percentage agreement %	*κ* value (95% CI)
Serum^*a*^	42	92	0.56 (0.38–0.73)
Bronchoalveolar lavage^*b*^	14/19	70/71	0.18 (−0.14–0.50)/0.24 (−0.07–0.55)
Other respiratory specimens^*b*^	26/37	35/37	0.17 (0.04–0.30)/0.22 (0.08–0.37)
Bronchial aspirates^*b*^	21/32	39/41	0.15 (−0.03–0.33) 0.22 (0.03–0.40)
TAs^*b*^	32/42	30/32	0.18 (−0.03–0.38) 0.22 (−0.01–0.44)

^*a*^ Platelia cut-off value: ≥0.5.

^*b*^ Platelia cut-off value: ≥1/≥0.5.

We next compared the qualitative performance of both GLM immunoassays according to patient group. There was a sufficient number of specimens collected from hematological patients to conduct a separate analysis (296 specimens from 72 patients). This was deemed of clinical interest due to the particular nature of these patients (i.e., use of antifungal prophylaxis and concurrence of several IPA risk factors). The data roughly reproduced that observed for the entire cohort and the other two patient groups analyzed separately (Table S3). First, overall, less than one-third of specimens testing positive with the Virclia assay returned positive results with the Platelia assay (regardless of the index value cut-off used for respiratory specimens). Second, the percentage of specimens yielding Virclia positive/Platelia negative results was noticeably higher for respiratory specimens (100% for BAL and around 70% for other respiratory specimens) than for sera (62%). Accordingly, the level of agreement between assays was substantially higher for sera (*κ* index, 0.47) than for LRT specimens (*κ* values ≤ 0.13) (Table S4).

A total of 58 paired BAL and BAS specimens (collected in the same bronchoscopy procedure) were available from 53 patients. As shown in Table 4 a higher rate of positive results was obtained in BAS than in BAL specimens, regardless of the GLM immunoassay used (the index value cut-off for positivity of the Platelia assay was set at ≥0.5 in this analysis). The difference between positive yields in BAS vs BAL specimens was particularly marked for the Virclia assay (15 BAS positive/BAL negative and only 2 BAS negative/BAL positive).

**TABLE 4:** Qualitative results returned by the Virclia Monotest and the Platelia Aspergillus Antigen assay in paired BAs and bronchoalveolar lavages

Results returned by GLM immunoassays in BAL specimens^*a*^	Results returned by GLM immunoassays in BAS specimens^*a*^				
	Virclia			Platelia	
	Positive	Negative	Indeterminate	Positive	Negative
Virclia Positive	14	2	2	–^*b*^	–
Virclia Negative	15	14	6	–	–
Virclia Indeterminate	4	1	0	–	–
Platelia Positive	–	–	–	3	1
Platelia Negative	–	–	–	5	49

^*a*^ Aspergillus GLM antigen Virclia Monotest results: positive values ≥0.2; negative values <0.2; Indeterminate values between 0.16 and 0.19. Platelia Aspergillus Antigen Assay result: Positive ≥0.5 in serum samples and lower respiratory tract specimens.

^*b*^ NA, not applicable.

### Clinical performance of the galactomannan immunoassays

The patient with proven IPA was categorized as concordant because both GLM immunoassays returned at least one positive result (regardless of the specimen type). Regarding the 11 patients with probable disease, 10 were deemed to be concordant and 1 discordant (Virclia positive/Platelia negative). When considering the patients with no IPA diagnosis (*n* = 132), there were 96 patients categorized as concordant (84 Virclia negative/Platelia negative, 12 Virclia positive/Platelia positive) and 36 discordant (34 Virclia positive/Platelia negative and 2 Platelia positive/Virclia negative). The overall sensitivity and specificity of the Virclia assay for the diagnosis of proven/probable IPA were 100% (95% CI, 75.7%–100%) and 65% (95% CI, 56.7%–72.7%), respectively, and for the Platelia immunoassay 91.7% (95% CI, 64.6%–98.5%) and 89.4% (95% CI, 83.0%–93.6%), respectively (*P* ≤ 0.001 for clinical specificity). Overall (all specimens considered together), as shown in Fig. S1, the areas under the ROC curves (AUCs) were nevertheless comparable (0.85 for the Virclia test and 0.79 for the Platelia assay; *P* = 0.21). Likewise, as shown in Fig. S2, comparable AUCs were observed for sera (Virclia assay: 0.80; 95% CI, 0.75–0.85 vs Platelia assay: 0.87; 95% CI, 0.82–0.91; *P* = 0.23), combined sera and BAL (Virclia assay: 0.81; 95% CI, 0.76–0.85 vs Platelia assay: 0.88; 95% CI, 0.84–0.92; *P* = 0.16) and other respiratory specimens (BAS and TA) (Virclia assay: 0.81; 95% CI, 0.72–0.88 vs. Platelia assay: 0.74; 95% CI, 0.65–0.82; *P* = 0.28).

### Quantitative correlation between index values returned by the galactomannan immunoassays

We first assessed the correlation between quantitative values provided by both immunoassays using the Aspergillus Galactomannan VIRCLIA sample set. All vials containing lyophilized material were reconstituted with distilled water following the manufacturer’s recommendation and assayed by both platforms in duplicate. The data are shown in Table S2. All negative samples (*n* = 10) were correctly categorized as such by the Virclia assay, whereas one returned a positive result with the Platelia assay (S9). In turn, all positive samples (*n* = 14) yielded positive results with both GLM immunoassays. The index values returned by both immunoassays were optimally correlated (*ρ* = 0.97; 95% CI, 0.93–0.98; *P* < 0.001). We next made up a sample set consisting of GLM-negative pooled sera, BAL or TA spiked with known GLM concentrations ranging from 0.0015 to 0.1 µg/mL. Spiked specimens were pretreated and run (in duplicate) in parallel with both immunoassays. Data are shown in Table S5. GLM was detected in spiked serum and BAL matrices when the GLM concentration was ≥0.0125 µg/mL and the positive cut-off for the Platelia assay set at ≥0.5. Lower GLM concentrations were, however, inconsistently detected in these specimens by both immunoassays. Interestingly, lower concentrations (0.0015 µg/mL) of GLM were detected in spiked TA by both immunoassays. Both assays exhibited linearity across the GLM concentrations tested in spiked serum and BAL; this was not the case for spiked TA for GLM concentrations ≥ 0.050 µg/mL.

**FIG 2:**
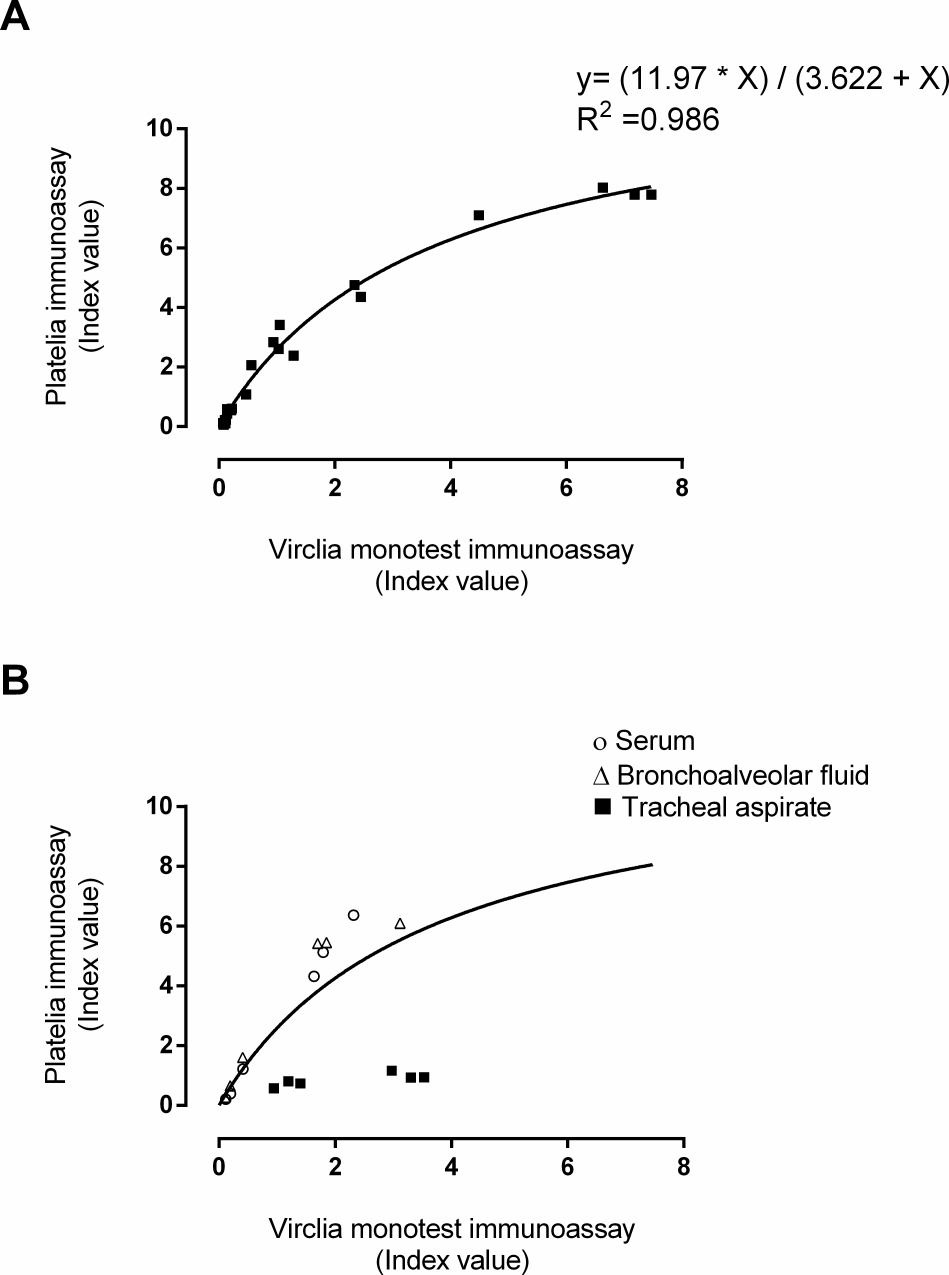
Conversion of Virclia immunoassay index values into Platelia immunoassay index values either using the *Aspergillus* Galactomannan VIRCLIA sample set (A) or serum, bronchoalveolar fluid, and TA spiked with known concentrations of the above standard (B). Index values represent means of duplicates.

**FIG 3:**
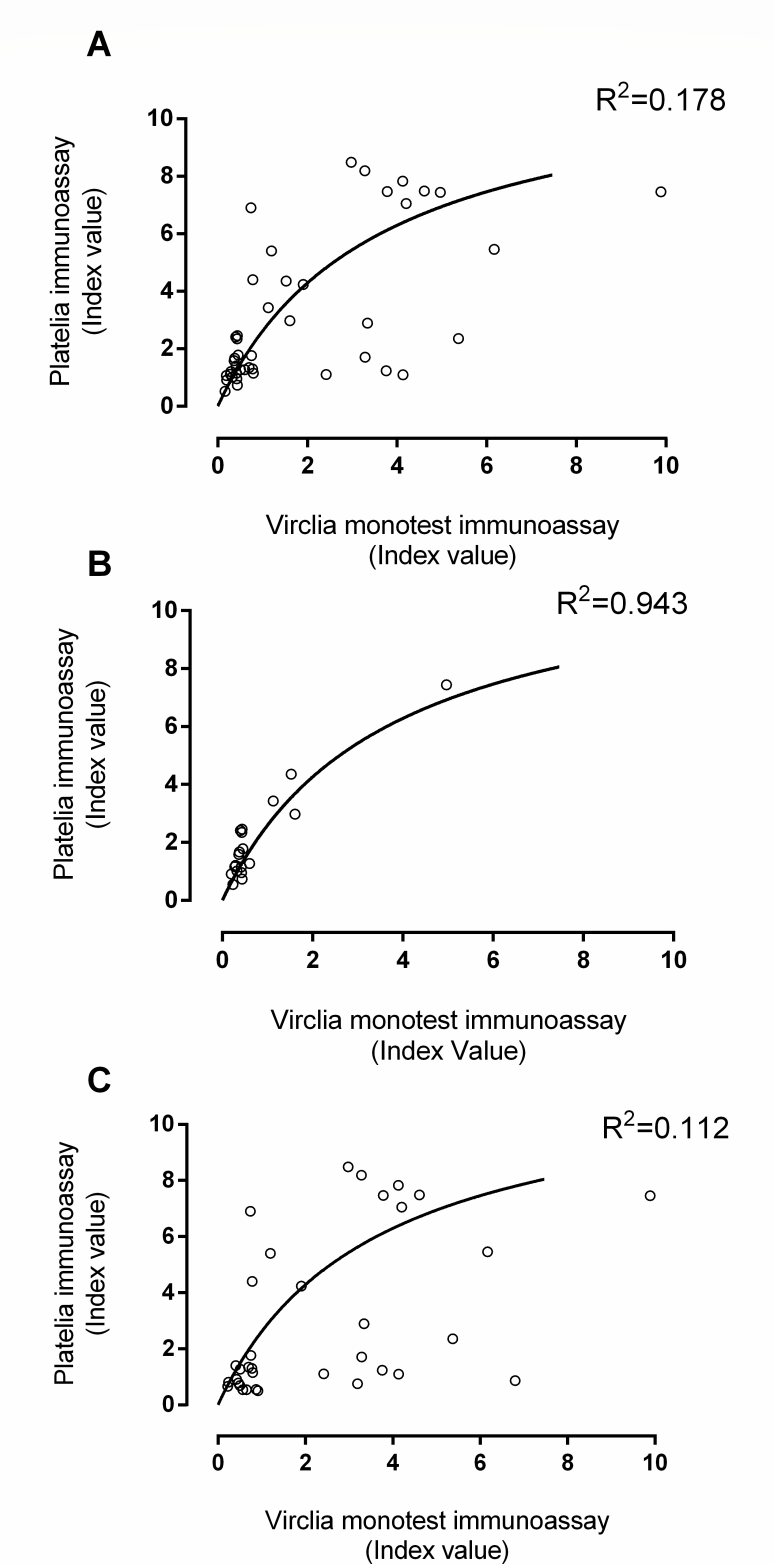
Fitting regression model of the results returned by the Virclia and Platelia immunoassays for all specimens included in the study (A), combined bronchoalveolar lavage and sera (B), and off-label specimens (BAs and TAs) in combination (C).

The correlation between index values in clinical samples returned by both GLM immunoassays was moderate (*ρ* = 0.64; 95% CI, 0.41–0.78; *P* < 0.001) when all specimens were considered in the analysis; nevertheless, it was strong for on-label samples (*ρ* = 0.73; 95% CI, 0.42–0.89; *P* < 0.001). The correlation index decreased (Rho = 0.52; 95% CI, 022–0.73; *P* = 0.001) when only BAS and TA were selected (in combination) for the analysis. The conversion of Virclia index values into Platelia index values using the Aspergillus Galactomannan VIRCLIA sample set could be derived via the following formula *y* = (11.97 * *X*)/3.62 + *X*), as shown in Fig. 2 (panel A). Data from spiked serum and BAL fitted well with the regression model, whereas data from TA did not (Fig. 2, panel B). Regarding clinical specimens testing positive in both GLM immunoassays, as shown in Fig. 3, serum/BAL clinical specimens optimally fitted the regression model (*R*^2^ = 0.94), whereas all specimens in combination and off-label specimens (BAS and TA) did not (*R*^2^ = 0.17 and *R*^2^ = 0.11, respectively). The figures for GLM positive and negative specimens were as follows: *R*^2^ = 0.50 for serum/BAL specimens; *R*^2^ = 0.16, for all specimens; *R*^2^ = 0.10 for off-label specimens).

## DISCUSSION

The Platelia Aspergillus Antigen immunoassay is nowadays the “gold standard” for Aspergillus GLM measurements in serum and BAL specimens; it is thus instrumental for the diagnosis of IPA in a variety of clinical settings (1, 6–9). The Virclia GLM immunoassay is rapid, easy to carry out, and can be performed in a monotest format, thus avoiding batch testing, which is a routine practice in most centers using the Platelia assay. Positioning of Virclia immunoassays as a mycological criterium for IPA requires a thorough assessment of its analytical and clinical performance compared to that of the Platelia assay, which was our aim in this study. To our knowledge, only three studies have previously addressed this goal (18–20). Buil *et al.* (20), enrolled patients with hematological disease and compared the performance of both immunoassays in BAL specimens. In turn, Troncoso *et al.* (18) and Leyva Calero *et al.* (19), in their analyses included BAL and sera collected from patients with different clinical conditions. Our study included prospectively collected consecutive specimens from patients with a wide variety of clinical conditions tested for GLM upon clinical request, allowing us to compare the performance of both methods in a “real-life setting”. In contrast, a prospective/retrospective study design was used in previous studies (19, 20), resulting in cohorts “enriched” in proven and probable IA cases. Thus, comparison across these studies (18–20) and between them and ours is not straightforward. Our study was singular; in that, it included off-label LRT samples for both immunoassays (BAS and TA). We found this approach of interest given that TA are frequently sampled in mechanically ventilated patients for microbiological studies and that BAS is a more “concentrated” and rather more homogeneous specimen than BAL, the latter because of the instillation and suction volumens may differ across centers.

Our data indicated that the Virclia assay returns more positive results than the Platelia assay in all specimen types assayed; consequently, the PPA across assays was low overall. As expected, median Virclia index values of specimens testing negative with the Platelia assay were significantly lower than those testing positive. In contrast, the number of specimens testing positive with Platelia and negative with Virclia was negligible. These data concur with that previously reported for on-label specimens (18–20). Of relevance, the degree of qualitative agreement across the assays, as assessed by Kappa statistics, was overall much higher for sera (0.56) than for BAL (≤0.24), as previously reported (10), or for BAS and TA (≤0.22). The same observation was made when hematological patients were analyzed separately. Leyva Calero *et al.* (19) reported a *κ* value of 0.72, considering sera and BAL in combination for the analysis. Potentially relevant differences between our study and theirs (19) include the number of patients with proven/probable IPA in the cohorts, patients’ clinical characteristics, the consideration of Virclia indeterminate results (excluded from the analyses herein) for analyses, and, importantly, the number of specimens per patient collected, which was more than one in around two-thirds of patients in our cohort and a single sample in the other studies (19, 20). In turn, the overall agreement reported by Buil *et al.* (20) for both immunoassays using BAL specimens collected from hematological patients was between 0.62 and 0.77, depending on the cut-off used for positivity (for both Virclia and Platelia). Unfortunately, no conclusion could be derived from the null agreement seen in the current study across both assays for BAL specimens from hematological patients, as there were very few of these in our sample set and none tested positive with the Platelia assay.

We next assessed the clinical diagnostic performance of both GLM assays. We reasoned that for this analysis, possible IPA cases had to be excluded, as some of which would have to be recategorized attending Platelia results; this would have introduced a clear bias skewed toward the Platelia assay. Thus, only patients with proven and probable IPA and controls (those in which IPA was ruled out) were selected for analysis. Unfortunately, this analysis was hampered by the limited number of cases, which also precluded performing subanalyses exclusively including patients of different clinical conditions or excluding off-label specimens. In this setting, the overall clinical sensitivity of the Virclia assay was higher than that of the Platelia assay (100% and 91.7%, respectively); the reverse was true regarding specificity (65% vs 89.4%, respectively). Nevertheless, the areas under the ROC curves (AUCs) were comparable (*P* = 0.21). Likewise the AUCs for both GLM immunoassays were comparable when the data were analyzed according to the sample type (sera, sera plus BAL or off-label specimens. Unfortunately, due to the very limited number of proven/probable IPA cased in the cohort, the study was not sufficiently powered to compare the clinical performance of both assays. The clinical sensitivity reported for the Virclia and Platelia assays in previous studies was 80.9% and 63.2%, respectively, in Leyva Calero *et al.* (19), and between 50% and 75% depending upon the criteria set for positivity for both assays in Buil *et al.* (20). In both studies, the specificity of both assays was high (above 90%) when probable/proven IPA cases were compared to non-IPA cases. Of interest, resetting the Virclia cut-off for positivity did not result in improved clinical performance (data not shown).

From a quantitative standpoint, by using the Aspergillus Galactomannan VIRCLIA sample set, we showed that the index values returned by both GLM immunoassays were optimally correlated (rho = 0.97). The degree of correlation was lower for on-label clinical samples (*ρ* = 0.73), a figure similar to that reported by Leyva Calero *et al.* (19) using sera and BAL in combination (*ρ* = 0.71) and by Buil *et al.* (20) employing just BAL specimens (*ρ* = 0.72). Importantly, the degree of correlation between index values returned by both assays was reported to be higher in sera than in BAL (0.88 vs 0.63) (20). Here, the correlation between index values obtained from both GLM assays substantially decreased when only BAS/TA specimens were considered for analysis (*ρ* = 0.52).

Interestingly, based upon GLM measurements from both immunoassays made with the Aspergillus Galactomannan VIRCLIA sample set, we built a regression model that allowed the conversion of Virclia index values to Platelia index values, defined by the formula *y* = (11.97 * *X*)/3.62 + *X*. Data on either GLM spiked or GLM-positive clinical serum and BAL specimens (considered in combination) fitted well to the model, whereas that of BAS/TA did not fit so well, perhaps due, at least in part, to the apparent lack of linearity of GLM measurements in these matrices above a certain GLM concentration.

The real-life prospective design is a major strength of the current study, which also had several limitations. First, the scarce number of proven and probable IPA cases in our cohort limited the soundness of the clinical sensitivity and specificity analyses. Second, we lacked comprehensive data collection regarding the use of drugs that may cause false-positive GLM results or antifungal prophylaxis/treatment, which may result in false-negative results. This is a drawback shared by the other studies mentioned above (18–20). Third, one could argue against our approach of eliminating possible IPA cases for clinical sensitivity/specificity analyses, and excluding the results of the Platelia assay for patient categorization. Nevertheless, Buil *et al.* (20) showed that the clinical sensitivity/specificity of both assays was quite comparable, irrespective of whether or not the Platelia GLM results were taken into consideration for patient categorization. We felt that by exclusively including proven/probable IPA cases we would increase the robustness of the analysis. Fourth, we included off-label (for both assays) BAS/TA specimens in our sample set. Leaving aside the potential clinical relevance of a positive result in BAS/TA, we proved these specimen types to be suitable for qualitative GLM detection; in fact, the limit of detection of both assays in TA, as deduced from experiments using the Aspergillus Galactomannan sample set, appeared to be even lower than in serum and BAL samples. Data on clinical specimens seemed to support this assumption, as the rate of positive results obtained with both GLM immunoassays in BAS compared with paired BAL specimens was much higher. Further studies are nevertheless required to assess the performance of the GLM immunoassays in these specimen types. Fifth, a non-validated sample set (Aspergillus Galactomannan VIRCLIA sample set) was used as a standard for certain experiments. Sixth, indeterminate results returned by the Virclia assay were eliminated rather than interpreted as positive or negative from the data set for analyses. Seventh, serial samples from more than half the patients in our cohort were available for analysis. We assume that these were collected under different clinical and therapeutic circumstances (i.e., pre- or post-IPA diagnosis or pre- or post-administration of antifungal drugs), which could have a differential impact on the performance of the two GLM immunoassays.

Because of the consolidated use of the Platelia assay in clinical practice, and to certain reluctance of clinicians to modify long-established guidelines, our model allowing the conversion of Virclia index values into Platelia values may contribute toward positioning the Virclia assay within the diagnostic algorithm of IPA. Our data warrant further prospective well-powered studies designed to assess the clinical performance of the Virclia GLM immunoassay in homogeneous clinical settings, not only using classic specimens, such as serum and BAL but also samples such as TA, which are commonly processed for microbiological studies in ICU patients undergoing mechanical ventilation.

## SUPPLEMENTAL MATERIAL

Figure S1 spectrum.03910-23-s0001.tif Receiver operating characteristic (ROC) curves of the Virclia and Platelia immunoassays.

Figure S2 spectrum.03910-23-s0002.tif Receiver operating characteristic (ROC) curves of the Virclia and Platelia immunoassays.

Table S1 spectrum.03910-23-s0003.docx Distribution of specimens collected from patients included in the study.

Table S2 spectrum.03910-23-s0004.docx Analysis of the *Aspergillus* Galactomannan VIRCLIA.

Table S3 spectrum.03910-23-s0005.docx Qualitative agreement between results returned by the Platelia *Aspergillus* Antigen and *Aspergillus* Galactomannan antigen.

Table S4 spectrum.03910-23-s0006.docx Positive and negative percentage agreement and Kappa coefficient of agreement.

Table S5 spectrum.03910-23-s0007.docx Analysis of *Aspergillus* Galactomannan spiked specimens.

ASM does not own the copyrights to Supplemental Material that may be linked to, or accessed through, an article. The authors have granted ASM a non-exclusive, world-wide license to publish the Supplemental Material files. Please contact the corresponding author directly for reuse.
