# Supplementary figures and images for: Comparative performance of the Platelia Aspergillus Antigen and Aspergillus Galactomannan antigen Virclia Monotest immunoassays in serum and lower respiratory tract specimens: a “real-life” experience
Source: Microbiol Spectr. 2024 Jun 25;12(8):e03910-23. doi: 10.1128/spectrum.03910-23 (PMC11302238; doi:10.1128/spectrum.03910-23)

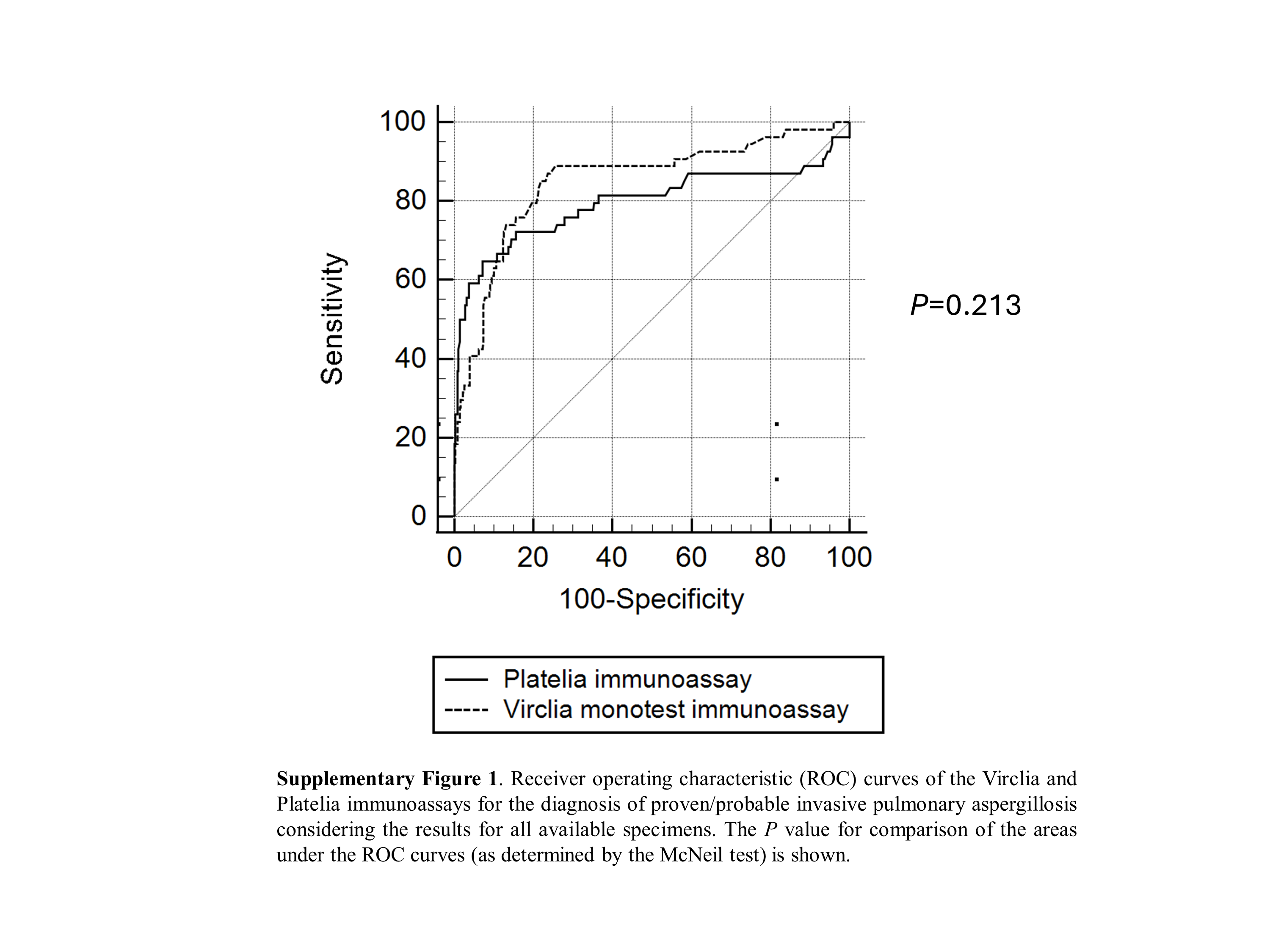

Supplement: Figure S1 — Receiver operating characteristic (ROC) curves of the Virclia and Platelia immunoassays. [file spectrum.03910-23-s0001.tif]

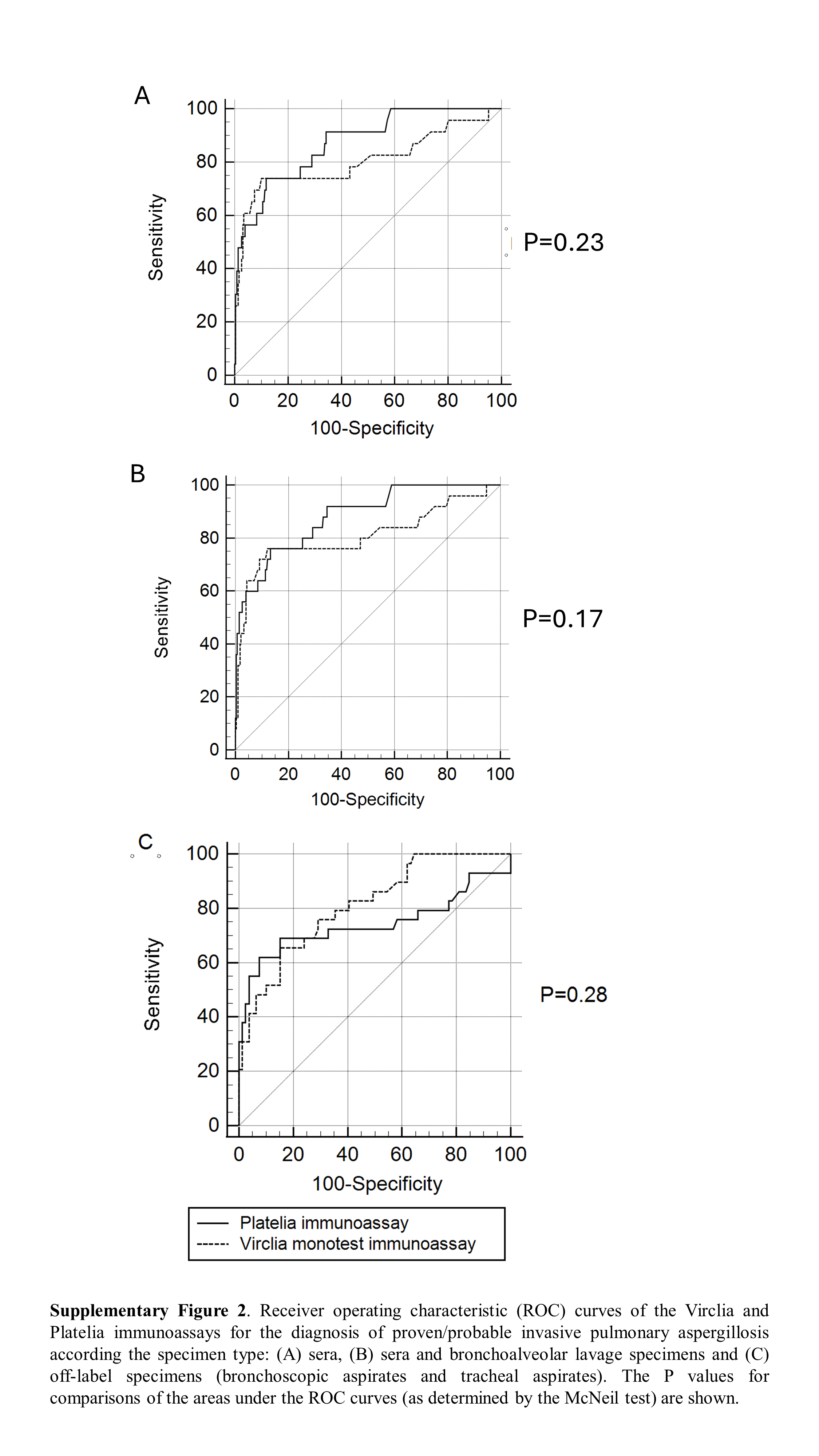

Supplement: Figure S2 — Receiver operating characteristic (ROC) curves of the Virclia and Platelia immunoassays. [file spectrum.03910-23-s0002.tif]
