# Supplementary material for: Comparative performance of the Platelia Aspergillus Antigen and Aspergillus Galactomannan antigen Virclia Monotest immunoassays in serum and lower respiratory tract specimens: a “real-life” experience
Source: Microbiol Spectr. 2024 Jun 25;12(8):e03910-23. doi: 10.1128/spectrum.03910-23 (PMC11302238; doi:10.1128/spectrum.03910-23)
Supplement: Table S1 — Distribution of specimens collected from patients included in the study. [file spectrum.03910-23-s0003.docx]

| **Supplementary Table 1. Distribution of specimens collected from patients included in the study.** | | |
| --- | --- | --- |
| No. of specimens per patient (no. of patients) | Specimen type | No. of patients |
| 1 (65) | Serum | 47 |
|  | BAL | 5 |
|  | BAS | 6 |
|  | TA | 7 |
| 2 (43) | Serum x 2 | 13 |
|  | Serum+ BAL | 2 |
|  | Serum + BAS | 5 |
|  | BAL + BAS | 23 |
| 3 (29) | Serum x 3 | 5 |
|  | Serum x 2 + TA | 2 |
|  | Serum + TA x 2 | 2 |
|  | Serum x 2 + BAS | 1 |
|  | Serum + BAS x 2 | 18 |
|  | TA x 3 | 1 |
| 4 (14) | Serum x 4 | 3 |
|  | Serum x 2 + BAS + TA | 2 |
|  | Serum x 2 + BAS x 2 | 1 |
|  | Serum + BAL x 2 + BAS | 1 |
|  | Serum x 2 + TA x 2 | 1 |
|  | Serum x 2 + BAS x 2 | 2 |
|  | Serum + BAS + TA x 2 | 1 |
|  | Serum + TA x 3 | 2 |
|  | BAL x 2 + BAS x 2 | 1 |
| 5 (5) | Serum + TA x 4 | 1 |
|  | Serum x 5 | 2 |
|  | Serum x 2 + BAL + BAS x 2 | 1 |
|  | BAL x 2 + BAS x 2 + Serum | 1 |
| 6 (4) | Serum x 6 | 1 |
|  | Serum x 2 BAL x 2 + BAS x 2 | 1 |
|  | Serum x 3 + TA x 2 + BAL | 1 |
|  | Serum x 2 + BAS x 4 | 1 |
| 7 (1) | Serum x 7 | 1 |
| 8 (2) | Serum x 8 | 2 |
| 9 (2) | Serum x 1 + TA x 8 | 1 |
|  | BAS x 3 + BAL x 4 + Serum x 2 | 1 |
| 10 (4) | Serum x 10 | 2 |
|  | Serum x 8 + TA x 2 | 1 |
|  | Serum x 4 + TA x 4 + BAS x 2 | 1 |
| 11 (3) | Serum x 11 | 1 |
|  | Serum x 5 + BAL x 2 + BAS x 4 | 1 |
|  | Serum x 4 + BAL + BAS + TA x 5 | 1 |
| 12 (1) | Serum x 12 | 1 |
| 13 (1) | Serum x 13 | 1 |
| 15 (1) | Serum x 15 | 1 |
| 18 (1) | Serum x 10 + BAL x 3 + BAS x 2 + TA x 3 | 1 |
| 20 (1) | Serum x 18 + BAL x 1 + BAS x1 | 1 |
| BAL, bronchoalveolar lavage, BAS, bronchoscopic aspirate; TA, tracheal aspirate. | | |
