# Supplementary material for: Comparative performance of the Platelia Aspergillus Antigen and Aspergillus Galactomannan antigen Virclia Monotest immunoassays in serum and lower respiratory tract specimens: a “real-life” experience
Source: Microbiol Spectr. 2024 Jun 25;12(8):e03910-23. doi: 10.1128/spectrum.03910-23 (PMC11302238; doi:10.1128/spectrum.03910-23)
Supplement: Table S2 — Analysis of the Aspergillus Galactomannan VIRCLIA. [file spectrum.03910-23-s0004.docx]

| **Supplementary Table 2. Analysis of the Aspergillus Galactomannan VIRCLIA® sample set by the Platelia Aspergillus Antigen and Aspergillus Galactomannan antigen Virclia Monotest immunoassays** | | | |
| --- | --- | --- | --- |
| Aspergillus Galactomannan VIRCLIA® sample set^a^ | Galactomannan concentration  (µg/mL) | Virclia index value | Platelia index value |
| S1 | 0.1 | 1.04 | 3.41 |
| S2 | 0 | 0.14 | 0.43 |
| S3 | 1 | 7.47 | 7.79 |
| S4 | 0 | 0.07 | 0.12 |
| S5 | 0.085 | 0.94 | 2.84 |
| S6 | 0.035 | 4.49 | 7.10 |
| S7 | 0 | 0.08 | 0.11 |
| S8 | 0.75 | 7.18 | 7.80 |
| S9 | 0.0075 | 0.21 | 0.55 |
| S10 | 0 | 0.14 | 0.58 |
| S11 | 0.045 | 0.56 | 2.06 |
| S12 | 0 | 0.10 | 0.22 |
| S13 | 0 | 0.11 | 0.12 |
| S14 | 0.125 | 1.29 | 2.38 |
| S15 | 0.015 | 0.21 | 0.61 |
| S16 | 0.17 | 2.34 | 4.75 |
| S17 | 0 | 0.10 | 0.10 |
| S18 | 0.03 | 0.47 | 1.08 |
| S19 | 0.5 | 6,63 | 8.02 |
| S20 | 0 | 0,08 | 0.06 |
| S21 | 0.06 | 1.03 | 2.61 |
| S22 | 0 | 0.10 | 0.08 |
| S23 | 0 | 0.12 | 0.23 |
| S24 | 0.25 | 2.45 | 4.36 |
| ^a^Samples were reconstituted with distilled water and assayed in duplicate (mean values are shown). | | | |
