# Supplementary material for: Comparative performance of the Platelia Aspergillus Antigen and Aspergillus Galactomannan antigen Virclia Monotest immunoassays in serum and lower respiratory tract specimens: a “real-life” experience
Source: Microbiol Spectr. 2024 Jun 25;12(8):e03910-23. doi: 10.1128/spectrum.03910-23 (PMC11302238; doi:10.1128/spectrum.03910-23)
Supplement: Table S3 — Qualitative agreement between results returned by the Platelia Aspergillus Antigen and Aspergillus Galactomannan antigen. [file spectrum.03910-23-s0005.docx]

| **Supplementary Table 3. Qualitative agreement between results returned by the Platelia Aspergillus Antigen and Aspergillus Galactomannan antigen Virclia Monotest according to the patient group and sample type** | | |
| --- | --- | --- |
| Aspergillus Galactomannan antigen Virclia Monotest result according to sample type (no. of specimens)^a^ | Platelia Aspergillus Antigen assay result^b^ | |
|  | Positive  (for lower respiratory tract specimens ≥1/≥0.5) | Negative  (for lower respiratory tract specimens <1/<0.5) |
| **Hematological patients** | | |
| **All specimens (n=296)** | | |
| Positive (47) | 10/11 | 37/36 |
| Negative (232) | 2/4 | 230/228 |
| Indeterminate (17) | 0/1 | 17/16 |
| **Sera (n=229)** |  |  |
| Positive (16) | 6 | 10 |
| Negative (200) | 2 | 198 |
| Indeterminate (13) | 1 | 12 |
| **Bronchoalveolar lavage (n=28)** |  |  |
| Positive (8) | 0/0 | 8/8 |
| Negative (19) | 0/0 | 19/19 |
| Indeterminate (1) | 0/0 | 1/1 |
| **Other respiratory specimens (37)^c^** |  |  |
| Positive (21) | 4/5 | 19/18 |
| Negative (13) | 0/2 | 13/11 |
| Indeterminate (3) | 0/0 | 3/3 |
| **ICU patients** | | |
| **All specimens (n=100)** | | |
| Positive (62) | 24/28 | 38/34 |
| Negative (30) | 0/0 | 30/30 |
| Indeterminate (8) | 0/0 | 8/8 |
| **Sera (n=38)** | | |
| Positive (15) | 8 | 7 |
| Negative (19) | 0 | 19 |
| Indeterminate (4) | 0 | 4 |
| **Bronchoalveolar lavage (n=3)** | | |
| Positive (2) | 2/2 | 0/0 |
| Negative (0) | 0/0 | 0/0 |
| Indeterminate (1) | 0/0 | 1/1 |
| **Other respiratory specimens (59)^c^** | | |
| Positive (45) | 14/18 | 31/27 |
| Negative (11) | 0/0 | 11/11 |
| Indeterminate (3) | 0/0 | 3/3 |
| **Hospitalized patients (other wards)** | | |
| **All specimens (n=139)** | | |
| Positive (42) | 10/17 | 32/25 |
| Negative (84) | 2/2 | 82/82 |
| Indeterminate (13) | 1/1 | 12/12 |
| **Sera (n=53)** | | |
| Positive (3) | 2 | 1 |
| Negative (46) | 2 | 44 |
| Indeterminate (4) | 0 | 4 |
| **Bronchoalveolar lavage (n=39)** | | |
| Positive (11) | 1/2 | 10/9 |
| Negative (23) | 0/0 | 23/23 |
| Indeterminate (5) | 1/1 | 4/4 |
| **Other respiratory specimens (47)^c^** | | |
| Positive (28) | 7/13 | 21/15 |
| Negative (15) | 0/0 | 15/15 |
| Indeterminate (4) | 0/0 | 4/4 |
| ^a^Aspergillus Galactomannan antigen Virclia Monotest results: positive values ≥0.2; negative values <0.2; Indeterminate values between 0.16-0.19.  ^b^Platelia Aspergillus Antigen Assay result: Positive ≥0.5 in serum samples and either ≥0.5 or ≥1 in lower respiratory tract specimens.  ^c^Bronchoscopic aspirates (n=33); Tracheal aspirates (n=4). | | |
