# Supplementary material for: Comparative performance of the Platelia Aspergillus Antigen and Aspergillus Galactomannan antigen Virclia Monotest immunoassays in serum and lower respiratory tract specimens: a “real-life” experience
Source: Microbiol Spectr. 2024 Jun 25;12(8):e03910-23. doi: 10.1128/spectrum.03910-23 (PMC11302238; doi:10.1128/spectrum.03910-23)
Supplement: Table S4 — Positive and negative percentage agreement and Kappa coefficient of agreement. [file spectrum.03910-23-s0006.docx]

| **Supplementary Table 4. Positive and negative percentage agreement and Kappa coefficient of agreement between qualitative results returned by the Platelia Aspergillus Antigen and Aspergillus Galactomannan antigen Virclia Monotest in specimens collected from hematological patients according to sample type** | | | |
| --- | --- | --- | --- |
| **Sample type** | **Positive percentage agreement %** | **Negative percentage agreement %** | **Kappa value (95% CI)** |
| Serum | 33 | 94 | 0.47 (0.18-0.76) |
| Bronchoalveolar lavage^a^ | - | 70 | 0.00 (-0.58-0.58) |
| Other respiratory specimens^b^ | 17/20 | 41/35 | 0.13 (-0.14-0.40)  0.05 (-0.23 0.33) |
| ^a^Platelia cut-off value: ≥0.5.  ^b^Platelia cut-off value: ≥1/≥0.5. | | | |
