# Supplementary material for: Comparative performance of the Platelia Aspergillus Antigen and Aspergillus Galactomannan antigen Virclia Monotest immunoassays in serum and lower respiratory tract specimens: a “real-life” experience
Source: Microbiol Spectr. 2024 Jun 25;12(8):e03910-23. doi: 10.1128/spectrum.03910-23 (PMC11302238; doi:10.1128/spectrum.03910-23)
Supplement: Table S5 — Analysis of Aspergillus Galactomannan spiked specimens. [file spectrum.03910-23-s0007.docx]

| **Supplementary Table 5. Analysis of Aspergillus Galactomannan spiked specimens with the Platelia Aspergillus Antigen and Aspergillus Galactomannan antigen Virclia Monotest immunoassays** | | | | | | |
| --- | --- | --- | --- | --- | --- | --- |
| Galactomannan concentration  (µg/mL)^a^ | Spiked serum | | Spiked Bronchoalveolar lavage | | Spiked tracheal aspirate | |
|  | Virclia  Index value | Platelia  Index value | Virclia  Index value | Platelia  Index value | Virclia  Index value | Platelia  Index value |
| 0.0015 | 0.12 | 0.21 | 0.10 | 0.27 | 0.94 | 0.57 |
| 0.0125 | 0.21 | 0.40 | 0.29 | 0.65 | 1.20 | 0.81 |
| 0.025 | 0.41 | 1.22 | 0.41 | 1.61 | 1.40 | 0.74 |
| 0.050 | 1.63 | 4.31 | 1.85 | 5.45 | 3.53 | 0.94 |
| 0.075 | 1.80 | 5.12 | 1.70 | 5.42 | 2.97 | 1.17 |
| 0.10 | 2.31 | 6.36 | 3.12 | 6.10 | 3.30 | 0.93 |
| ^a^Galactomannan-negative pooled sera, bronchoalveolar lavage or tracheal aspirates were spiked with known concentrations of galactomannan (*Aspergillus* Galactomannan VIRCLIA® sample set). Samples were assayed in duplicate (mean values are shown). | | | | | | |
